# Effects of Incretin Therapy on Skeletal Health in Type 2 Diabetes—A Systematic Review

**DOI:** 10.1002/jbm4.10817

**Published:** 2023-09-25

**Authors:** Rikke Viggers, Nicklas Højgaard‐hessellund Rasmussen, Peter Vestergaard

**Affiliations:** ^1^ Steno Diabetes Center North Denmark Aalborg Denmark; ^2^ Department of Endocrinology Aalborg University Hospital Aalborg Denmark

**Keywords:** analysis/quantitation of bone, animal models, bone histomorphometry, bone QCT/micro–CT, fracture risk assessment, practice/policy‐related issues

## Abstract

Diabetes poses a significant risk to bone health, with Type 1 diabetes (T1D) having a more detrimental impact than Type 2 diabetes (T2D). The group of hormones known as incretins, which includes gastric inhibitory peptide (GIP) and glucagon‐like peptide 1 (GLP‐1), play a role in regulating bowel function and insulin secretion during feeding. GLP‐1 receptor agonists (GLP‐1 RAs) are emerging as the primary treatment choice in T2D, particularly when atherosclerotic cardiovascular disease is present. Dipeptidyl peptidase 4 inhibitors (DPP‐4is), although less potent than GLP‐1 RAs, can also be used. Additionally, GLP‐1 RAs, either alone or in combination with GIP, may be employed to address overweight and obesity. Since feeding influences bone turnover, a relationship has been established between incretins and bone health. To explore this relationship, we conducted a systematic literature review following the PRISMA guidelines. While some studies on cells and animals have suggested positive effects of incretins on bone cells, turnover, and bone density, human studies have yielded either no or limited and conflicting results regarding their impact on bone mineral density (BMD) and fracture risk. The effect on fracture risk may vary depending on the choice of comparison drug and the duration of follow‐up, which was often limited in several studies. Nevertheless, GLP‐1 RAs may hold promise for people with T2D who have multiple fracture risk factors and poor metabolic control. Furthermore, a potential new area of interest is the use of GLP‐1 RAs in fracture prevention among overweight and obese people. Based on this systematic review, existing evidence remains insufficient to support a positive or a superior effect on bone health to reduce fracture risk in people with T2D. © 2023 The Authors. *JBMR Plus* published by Wiley Periodicals LLC on behalf of American Society for Bone and Mineral Research.

## Graphical abstract

Type 2 diabetes poses a significant risk to bone health. This systematic review reveals that incretin therapy remains insufficient to support a positive or superior effect on bone health to reduce fracture risk in people with T2D. Nevertheless, incretins may hold promise for people with T2D who have multiple fracture risk factors and poor metabolic control.

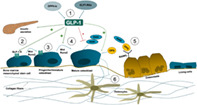

## Introduction

### Understanding the incretin system: implications for bone health

To comprehensively assess the impact of incretin therapy on bone health, it is crucial to grasp the functioning of incretins and their effects on the skeleton.

The incretins are a group of hormones released by the gastrointestinal tract in response to nutrient intake. Their primary role is to regulate insulin release in response to feeding, thereby modulating blood glucose levels.

#### The incretins comprise

1. Glucose‐dependent insulinotropic polypeptide, formerly known as gastric inhibitory peptide (GIP): GIP is secreted by the enteroendocrine K cells in the small intestine and has local inhibitory effects on gastric acid secretion. Moreover, GIP stimulates insulin secretion in a glucose‐dependent manner, contributing to the lowering of blood glucose.^(^ ^1^ ^)^
2. Glucagon‐like peptide 1 (GLP‐1) is secreted by the enteroendocrine L cells in the small and large intestines and is rapidly degraded by dipeptidyl peptidase 4 (DPP‐4). GLP‐1 stimulates insulin secretion in a glucose‐dependent manner and inhibits gastric emptying.^(^ ^2^ ^)^

Despite belonging to the glucagon superfamily, GLP hormones suppress endogenous glucagon secretion, further contributing to the glucose‐lowering effect.^(^ ^3^ ^)^

Diabetes is associated with compromised bone health, although recent studies have shown improvements in fracture risk.^(^ ^4^, ^5^ ^)^ The interplay between blood glucose, insulin, feeding, and bone health is complex.^(^ ^6^ ^)^

In general, eating suppresses bone resorption markers, especially C‐terminal telopeptide of collagen (CTX), although the effect on other bone markers such as tartrate‐resistant acid phosphatase type 5b (TRACP 5b) is smaller.^(^ ^7^, ^8^, ^9^ ^)^ A somewhat smaller effect of feeding is observed on formative markers, among others procollagen I N terminal propeptide (P1NP). The effect of feeding on bone turnover markers can be negated by the somatostatin analogue octreotide.^(^ ^10^ ^)^

In healthy control people, an oral glucose tolerance test (OGTT) suppresses CTX by approximately 50% compared to fasting levels. However, intravenous infusion of glucose keeping the same glucose levels as during the OGTT (isoglycemic intravenous glucose infusion [IIGI]) only marginally suppressed CTX (slightlymore than 30%) compared to the fasting state (a decline of around 30%). For P1NP no difference was present between IIGI and the fasting state, whereas the OGTT decreased P1NP by around 5%.^(^ ^11^ ^)^ GLP‐1 levels were similar during fasting and the IGII, whereas they—along with GIP—increased during the OGTT compared to the IGII and fasting.^(^ ^11^ ^)^ As a significant correlation was present between GIP and nadir CTX, these observations demonstrate an effect of incretins on bone turnover. GIP was higher during the IIGI than during fasting and lower during the OGTT.^(^ ^11^ ^)^ As mentioned earlier, octreotide, which inhibits incretins, negates the effects of the OGTT on bone turnover markers.^(^ ^10^ ^)^ A mixed meal test suppresses CTX by more than that observed during the OGTT.^(^ ^9^ ^)^ The suppression of P1NP is inversely dependent on insulin resistance, though this does not seem to be the case for CTX.^(^ ^9^ ^)^ Likewise, fasting P1NP seems to be inversely dependent on insulin resistance.^(^ ^9^ ^)^

Moreover, weight loss is a keystone in the prevention and treatment of Type 2 diabetes (T2D).^(^ ^12^ ^)^ However, weight loss is associated with bone loss measured by a decrease in bone mineral content (BMC) and bone mineral density (BMD) that is most likely a consequence of imbalances between bone resorption and formation.^(^ ^13^, ^14^, ^15^ ^)^ In people with T2D, a modest (and recommended) weight loss of approximately 7% over 1 year is found associated with a significant loss of bone mass that persists even if weight is maintained for the next 3 years.^(^ ^16^ ^)^ The loss of bone loss might also result in a further increased risk of fractures in these patients.^(^ ^17^ ^)^ The use of GLP‐1 RAs as treatment for T2D has emerged and shows beneficial aspects in both glycemic controls, as a weight loss facilitator, and in the prevention of other diabetes‐related comorbidities. Thus, it seems reasonable to investigate the potential impact on bone metabolism in people with T2D.

Collectively, incretin receptors are widely expressed, suggesting effects of incretin beyond the regulation of glucose homeostasis. Associations between incretin hormones and bone metabolism have emerged and opened up an interesting possibility of the interplay between feeding, obesity, T2D, and bone health. Frequent and excessive feeding may lead to prolonged suppression of bone turnover and increased insulin resistance. Likewise, significant weight loss might also induce a concomitant loss of bone mass. Consequently, incretin therapy may have the potential to impact bone health.

### Incretin therapy

In clinical practice, incretin therapy primarily involves the use of GLP‐1 RAs or DPP‐4is, commonly known as gliptins. There are several GLP‐1 RAs available, each with varying degrees of similarity to native GLP‐1 and different potencies for reducing HbA1c levels. Likewise, there are multiple DPP‐4is available on the market. Generally, DPP‐4is have a less potent glucose‐lowering effect compared to GLP‐1 RAs. However, unlike GLP‐1 RAs, which are typically administered via subcutaneous injections, DPP‐4is can be taken orally.

GLP‐1 RAs may be employed for the treatment of T2D, particularly in people with severe insulin resistance (SIRD) often associated with significant overweight.^(^ ^18^ ^)^ Notably, GLP‐1 RAs have also received therapeutic approval for managing obesity in the absence of diabetes.^(^ ^19^ ^)^ They can be used as monotherapy for weight management or in combination with GIP or amylin.^(^ ^20^, ^21^, ^22^ ^)^ Currently, the following GLP1‐RAs are approved by the US Food and Drug Administration and the European Medicines Agency for the treatment of T2D: dulaglutide, exenatide, liraglutide, lixisenatide, and semaglutide. Approved DPP‐4is are sitagliptin, saxagliptin, linagliptin, and alogliptin.^(^ ^23^ ^)^ Vildagliptin is only EMA approved. Tirzepatide is the first approved GIP and GLP1‐RA combination therapy for T2D. Only liraglutide and semaglutide are approved for treatment of obesity without existing diabetes.

### Diabetes and bone health

Type 1 diabetes (T1D) and T2D are associated with impaired bone health; in particular, a discrepancy between BMD and fracture risk has been reported.^(^ ^5^ ^)^ In general, people with T1D are more severely affected by lower BMD and higher fracture risk than people with T2D.^(^ ^5^ ^)^ In people with T2D, only some fracture types may be increased, whereas others may be lower than in the general population, perhaps due to a protective effect of being overweight.^(^ ^24^ ^)^

Incretins are mainly used to treat people with T2D and were recently upgraded from second‐line therapy in addition to or substituting metformin to a more prominent role in case of intended weight loss or presence of atherosclerotic cardiovascular disease.^(^ ^25^ ^)^ Incretins have also been used in T1D, although to a much lesser degree, and are not standard of care at present.

It is important to acknowledge that T2D encompasses various subtypes, ranging from the classic phenotype characterized by severe insulin resistance often associated with significant overweight and multiple diabetes‐related complications to milder forms such as obesity‐related (MOD) or age‐related (MARD) subtypes, as well as severe insulin‐deficient subtypes (SIDD).^(^ ^18^ ^)^ These different T2D phenotypes can have implications for bone health, considering that body weight itself is linked to fracture risk and BMD, as mentioned earlier. Therefore, when evaluating the choice of drug and comparator drugs, such as GLP‐1 RAs or DPP‐4is, these factors should be taken into consideration.^(^ ^26^ ^)^

## Effects of Incretins on Bone Health

### Methods

#### Literature survey

A systematic search was conducted on PubMed using specific free‐text search terms to gather available literature. The information base was updated with a cut‐off date of July 21, 2023. The three primary concepts were: fractures, bone turnover, and bone structure for GLP‐1 RAs and DPP‐4is. The three concepts were searched in isolation and then merged through the AND term with GLP‐1RA and DPP‐4i. The study types were organized into studies conducted on humans, animal studies, and cell studies. The screening process was carried out by the three coauthors (PV, NR, and RV), irrespective of language.

#### Search strategy

For GLP‐1 we initially used the search string “(fracture OR fractures) AND (‘GLP‐1 receptor agonist’ OR ‘GLP‐1 receptor agonists’ OR ‘glucagon‐like peptide 1 receptor agonist’ OR ‘glucagon‐like peptide 1 receptor agonists’),” which yielded 62 results. Then, secondly, the terms “(‘bone turnover’ OR ‘bone resorption’ OR ‘bone formation’) AND (‘GLP‐1 receptor agonist’ OR ‘GLP‐1 receptor agonists’ OR ‘glucagon‐like peptide 1 receptor agonist’ OR ‘glucagon‐like peptide 1 receptor agonists’)” were used, which yielded 38 hits. Third, “(‘bone structure’ OR ‘bone microstructure’ OR ‘bone mineral density’ OR ‘BMD’ OR ‘bone mineral content’ OR ‘bone strength’ OR ‘microindentation’) AND (‘GLP‐1 receptor agonist’ OR ‘GLP‐1 receptor agonists’ OR ‘glucagon‐like peptide 1 receptor agonist’ OR ‘glucagon‐like peptide 1 receptor agonists’)” yielded 26 results.

A similar search strategy was conducted for “DPP‐4is.” The first one on fractures yielded 68 results, including “(‘fracture’ OR ‘fractures’) AND (‘DPP‐4‐inhibitor’ OR ‘DPP‐4‐inhibitors’ OR ‘dipeptidyl peptidase‐4 inhibitor’ OR ‘dipeptidyl peptidase‐4 inhibitors’).” Secondly, bone turnover yielded 18 results using the terms “(‘bone turnover’ OR ‘bone resorption’ OR ‘bone formation’) AND (‘DPP‐4‐inhibitor’ OR ‘DPP‐4‐inhibitors’ OR ‘dipeptidyl peptidase‐4 inhibitor’ OR ‘dipeptidyl peptidase‐4 inhibitors’). Only one human study was available. Third was bone structure: “(‘bone structure’ OR ‘bone microstructure’ OR ‘bone mineral density’ OR ‘BMD’ OR ‘bone mineral content’ OR ‘bone strength’ OR ‘microindentation’) AND (‘DPP‐4‐inhibitor’ OR ‘dipeptidyl peptidase‐4 inhibitor’ OR ‘dipeptidyl peptidase‐4 inhibitors’),” which yielded 17 results.

#### Inclusion and exclusion

**Fig. 1:**
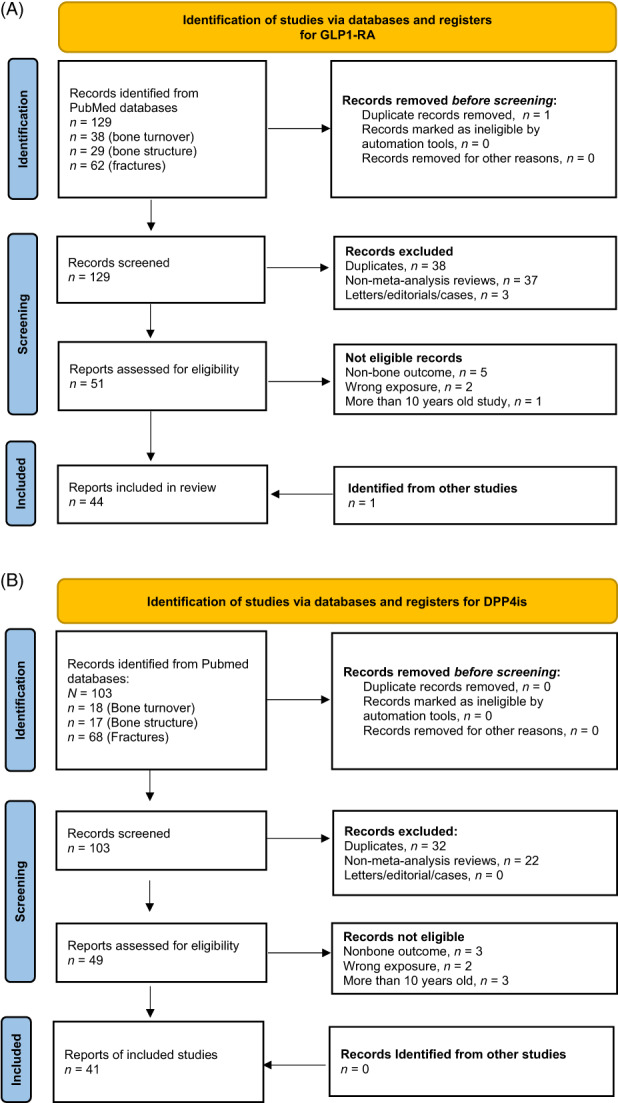
Flow diagrams. From: Page MJ, McKenzie JE, Bossuyt PM, Boutron I, Hoffmann TC, Mulrow CD, et al. PRISMA 2020 statement: an updated guideline for reporting systematic reviews. BMJ 2021;372:n71. doi: 10.1136/bmj.n71. For more information, visit: http://www.prisma‐statement.org/.

See Figure 1*A,B* (PRISMA flow) for flow of inclusion and exclusion of studies. Systematic reviews and meta‐analyses were given priority. If original studies were not included in the meta‐analyses, either due to oversight or being more recent than the meta‐analyses themselves, they were still cited. Older meta‐analyses were cited alongside more recent ones if there were discrepancies in their findings. If older meta‐analyses aligned with the newer ones, they were not cited. Additionally, other relevant articles referenced within the included records were reviewed for eligibility. While studies involving people with diabetes were prioritized, studies on nondiabetic people were also considered for comprehensive evaluation. Among studies involving humans, randomized controlled trials (RCTs) took precedence over observational studies. Regarding fractures, only human studies were considered eligible. It is worth noting that studies were often limited and frequently did not focus specifically on people with T2D. Finally, GLP‐1 RAs were given priority. Additionally, an overview of included studies regarding fracture risk, bone turnover, and bone structure for GLP‐1 RAs and DPP‐4is are presented in Tables 1, 2, 3, 4. Furthermore, some overlap was seen for eligibility between GLP‐1 RAs and DPP‐4is as several studies included both exposures. Hence, 44 studies were reviewed for GLP‐1RAs and 41 for DPP‐4is.

**Table 1:** Overview of Fracture Risk with Glucagon‐like Peptide 1 Receptor Agonists (GLP‐1 RAs) and Dipeptidyl Peptidase 4 Inhibitors (DPP‐4is)

Authors						
GLP‐1 RAs and fracture risk	Year	Purpose	Research design	Population (country, *n*)	Age (years)	Main outcomes
Al‐Mashhadi et al.	2022	Compare fracture risk among users of GLP‐1 RA and DDP‐4i as add‐on to metformin	Human, retrospective cohort study	Denmark, total *n* = 32,266 (ps. match 1:1), 43% female	56.6	No fracture risk difference between treatment with GLP‐1RA and DPP‐4i
Al‐Mashhadi et al.	2022	Compare fracture risk among users of GLP‐1 RA and SGLT2‐i as add‐on to metformin	Human, retrospective cohort study	Denmark, total *n* = 18,380 (ps. match 1:1); 40% female	60	SGLT2 inhibitors have no effect on fracture risk when compared to GLP‐1 receptor agonists
Zhuo et al.	2021	Compare fracture risk among users of GLP‐1 RA, DPP‐4i, and SGLT2‐i	Human, cohort, T2DM without previous fracture	Medicare Brigham, Boston, MA, USA. Type 2 diabetes, *n* = 137,667 (PS match 1:1:1)	>66	Risk of fractures did not differ between initiation of SGLT‐2i, DPP‐4i, or GLP‐1RA
Driessen et al.	2016	Examine fracture risk among users of GLP‐1 RA and DPP‐4i	Human meta‐analysis of four studies comparing fracture risk in GLP1‐RA and DPP4‐i	Four register‐based studies included; DPP4‐i users *n* = 22,961 and GLP‐1 RA users *n* = 8,505	N/A	Fracture risk not altered by incretin therapy
Driessen et al.	2015	Investigate association between GLP‐1 RA and risk of fracture	Human, case (fracture)‐control (no fracture) study	Denmark, sex‐ and age‐matched; cases (fracture) *n* = 229,145 matched to controls (no fracture) *n* = 229,145	55	The prior use of GLP‐1 RA was not higher among those with a fracture compared to controls, neither if stratified by GLP‐1 RA doses
Driessen et al.	2015	Investigate association between GLP‐1 RA and risk of fracture	Human, retrospective cohort study	UK, GLP1‐RA users *n* = 8,354 and never users *n* = 208,462; 47% female	53.5 and 61	GLP‐1 RA use did not affect fracture risk
Chai et al.	2022	Compare fracture risk among users of DPP‐4i, GLP‐1 RA, and SGLT2‐i compared with other antidiabetic agents or placebo	Human, network meta‐analysis	RCT studies (*n* = 177) with fracture outcome	N/A	Use of DPP‐4i, GLP‐1 RAs, or SGLT‐2i did not affect fracture risk in subjects with T2DM
Zhang et al.	2021	Evaluate effects of antidiabetic drugs on fracture risk in T2DM	Human meta‐analysis	RCT studies (*n* = 117)	N/A	Trelagliptin (RR 3.51; 1.58 to 13.70) increased the risk of fracture compared to placebo, whereas albiglutide (RR 0.29; 0.04 to 0.93) decreased the risk of fracture compared to placebo
Cheng et al.	2019	Evaluate association between GLP‐1 RAs and risk of bone fracture in T2DM	Human, meta‐analysis	RCT studies (*n* = 38)	N/A	Liraglutide and lixisenatide were associated with a fracture risk reduction compared to placebo and other antidiabetic drugs
Zhang et al.	2018	Analyze effects of GLP‐1 RA on fracture risk in T2DM	Human, network meta‐analysis.	RCT studies (*n* = 54)	N/A	GLP‐1 RAs were associated with decreased fracture risk compared to placebo or other antihyperglycemic drugs, and exenatide was associated with the lowest risk
Su et al.	2015	Evaluate risk of fractures associated with liraglutide or exenatide compared to placebo or other active drugs	Human, meta‐analysis	RCT studies, *n* = 16 (liraglutide *n* = 8, Exenatide *n* = 10)	N/A	Liraglutide was associated with a reduced risk of incident fractures and exenatide with an elevated risk
Mabilleau et al.	2014	Assess whether patients with T2D treated with GLP‐RAs present lower incidence of bone fracture compared with patients using other antidiabetic drugs	Human meta‐analysis	RCT studies (*n* = 28)	N/A	GLP‐1RAs does not modify risk of bone fracture in T2DM compared with the use of other antidiabetic medications

DPP‐4is and fracture risk						
Lui et al.	2023	Evaluate MOF risk among T2D patients treated with sodium‐glucose cotransporter‐2 inhibitors (SGLT2i) across eGFR and albuminuria categories	Human study: population‐based cohort study between 2007 and 2020; 1‐year follow‐up	*N* = 14,348 in each group of SGLT‐2 or DPP4‐inihibtors	60 ± 11 years	A cohort study on T2D subjects observed no statistically significant increase in fracture risk with SGLT2i use compared with DPP‐4 inhibitor use after 1 year
Tamaki et al.	2022	Clarify risks of initiating antidiabetic drugs for fractures	Human study: cohort study between 2012 and 2018	*N* = 966,700 outpatient participants initiating drug treatment for T2D	Patients aged ≥65 years	DPP‐4 inhibitor use was related to increased hip fracture risk compared to metformin; the risk of vertebral fractures was higher in subjects prescribed insulin, thiazolidine, and DPP‐4 inhibitors compared with metformin
Ohyama et al.	2023	Examine association between DPP4‐inhibitor use and risk of fracture in older patients	Human study: case–control study between 2004 and 2019	Japanese and U.S. FDA	N/A	Reduced fracture risk among those exposed to DPP‐4 inhibitors
Al Mashhadi et al.	2022	Investigate risk of MOFs for treatment with GLP‐1RA compared to DPP‐4is as add‐on therapies to metformin	Human study: population‐based cohort study	*N* = 32,266 individuals were included in main analysis (16,133 in each group)	57 ± 11 years	No significantly different risk of osteoporosis‐related fractures between subjects exposed to GLP‐1 RA and DPP‐4 inhibitors
Chang et al.	2022	Explore effect of DPP‐4i on risk of osteoporosis in Taiwanese patients with T2D	Human study: cohort study with 7 years of follow‐up	*N* = 6339 patients on DPP‐4i (DPP‐4i group) and *N* = 25,356 patients without DPP‐4i (non‐DPP‐4i group)	Mean age of 66 years	Significantly lower among subjects exposed to DPP‐4is compared with those without DPP‐4i treatment (adjusted HR 0.616; 95% CI 0.358 to 0.961; *p* = 0.011)
Ha et al.	2022	Determine whether SGLT2i were related to increased fracture risk in adults with T2D compared with DPP‐4i	Cohort study between 2016 and 2018	*N* = 478,826 new users of an SGLT2i or DPP‐4i matched 1:1	55 ± 12 years	SGLT2 inhibitor was not linked with increased fracture risk compared to DPP‐4is
Ustulin et al.	2019	Estimate effect of DPP‐4i on bone fracture risk in Korean population	Cohort study	Control group without diabetes (*n* = 5,582), treatment groups with diabetes included DPP‐4i users (*n* = 1,410) and DPP‐4i nonusers (*n* = 4,172)	N/A	No difference in fractures between DPP‐4i users and nonusers.
Cowan et al.	2022	Determine if SGLT2 inhibitor use associates with higher risk of fractures compared with DPP‐4is	Population‐based cohort study in Ontario, Canada between 2015 and 2019	*N* = 38,994 new users of a SGLT2 inhibitor and 37,449 new users of a DPP‐4i ≥66 years of age	N/A	Initiation of SGLT2 inhibitor versus a DPP‐4i was not associated with higher risk of fractures
Alkabbani	2023	Demonstrate impact of using active‐comparator‐restricted disproportionality analysis	Disproportionality analysis	Food and Drug Administration Adverse Event Reporting System	N/A	No differences in fracture risk among users of SGLT2 inhibitors versus DPP‐4is
Wang et al.	2022	Investigate longitudinal relationship of glycated hemoglobin (HbA1c) and common medications on fracture risk in people with T2D	Retrospective population‐based cohort between January 1, 2007, and September 30, 2015	*N* = 157,439 individuals with T2D aged ≥55 years	N/A	Treatment with DPP‐4is (HR: 0.93; 95% CI, 0.88 to 0.98) was associated with reduced all‐fracture risk
Patorno et al.	2022	Investigate effectiveness and safety outcomes among patients with T2D initiating empagliflozin versus DPP‐4i	Population‐based cohort study between 2014 and 2017	*N* = 39,072 pairs of 1:1 matched adult patients with T2D initiating empagliflozin or DPP‐4is	Mean age 60 years	Empagliflozin was associated with similar risk of fractures
Kong et al.	2021	Evaluate association between use of incretins and fracture risk	Update meta‐analyses	N/A	N/A	Incretins were not associated with fracture risk [0.97 (95% CI: 0.88 to 1.08)]; when sitagliptin 100 mg/day (OR 0.495, 95% CI: 0.304 to 0.806) or liraglutide 1.8 mg/day was administered (OR 0.621, 95% CI: 0.413 to 0.933), it reduced fracture risk
Han et. Al	2021	Examine real‐world cardiovascular effectiveness and safety associated with SGLT2 inhibitor compared with DPP‐4i treatment in T2D	Retrospective cohort study between September 2014 and December 2016	*N* = 408,506 new users of SGLT2 inhibitor or DPP‐4i were propensity score matched	73 ± 6 years	Risks of bone fracture were similar between both groups
Schmedt et al.	2019	Investigate whether use of SGLT‐2 inhibitors is associated with increased risk of fractures	Nested case–control between 2011 and 2016	*N* = 7,522 patients with fractures matched to 296,845 control subjects	66 ± 9 years	No risk of fracture found
Espeland et al.	2020	Compare cardiovascular safety of linagliptin with glimepiride in older and younger participants	Cohort study	N/A	40 to 85 years	Linagliptin had significantly lower risk of hypoglycemia and falls or fractures than glimepiride
Gamble et al.	2018	Determine association between DPP‐4is and common osteoporotic “fragility fractures”	Cohort study between 2007 and 2016	*N* = 7,993 and *N* = 26,636 new users of DPP‐4is and SUs, respectively	Mean age was 58.8 years	DPP‐4is were not associated with increased risk of fragility fractures compared with SUs or insulin
Hou et al.	2018	Investigate putative link between DPP‐4i use and risk of fracture in patients with T2D	Cohort study performed between 2009 and 2013	*N* = 3,996 patients with T2D used DPP‐4i as second‐line antidiabetic drug matched with controls 1:1	N/A	DPP‐4i usage was associated with reduced risk of fractures and upper extremity fractures in T2D
Yang et al.	2017	Systematically evaluate effects of DPP‐4is on bone fracture in T2D	Network meta‐analysis of randomized controlled trials	Studies: 75 RCTs with total of 70,207 patients	N/A	Risk of fracture for alogliptin tended to decrease compared to placebo (OR, 0.51; 95% CI, 0.29 to 0.88); in direct pairwise meta‐analysis, alogliptin was associated with nonsignificant tendency to fracture risk reduction compared with placebo (OR, 0.54; 95% CI, 0.29 to 1.01)
Hidayat et al.	2017	Identify risk of fracture in DPP4i use	Systematic review and meta‐analysis of observational studies	N/A	N/A	Use of DPP‐4is (RR 0.83, 95% CI, 0.60, 1.14; *n* = 11), GLP‐1 RA (RR 0.65, 95% CI, 0.24, 1.74; *n* = 4), or SGLT2 inhibitors (RR 1.02, 95% CI, 0.91, 1.16; *n* = 4) was not associated with risk of fracture

Abbreviation: MOF = major osteoporotic fracture; PS = propensity score; RCT = randomized controlled trial; SU = sulfonylurea; T2D = type 2 diabetes.

**Table 2:** Overview of Bone Turnover with Glucagon‐like Peptide 1 Receptor Agonists (GLP‐1 RAs) and Dipeptidyl Peptidase 4 Inhibitors (DPP‐4is)

Authors						
GLP‐1 RAs and bone turnover	Year	Purpose	Research design	Population (country, n)	Age (years)	Main outcomes
Human studies						
Hygum et al.	2019	Investigate effect of liraglutide on bone turnover in subjects with T2D	Human, RCT, liraglutide 1.8 mg daily or placebo for 26 weeks	Denmark, T2D patients, *n* = 60, liraglutide, *n* = 30 (14 female/16 male) and placebo, *n* = 30 placebo (16 female/14 male)	62 (liraglutide) and 64 (placebo)	No effect on CTX; P1NP increased after week 4; hip BMD decreased in placebo group and was unchanged in liraglutide group
Iepsen et al.	2015	Investigate role of liraglutide on bone formation and weight loss‐induced bone mass reduction	Human, 8 weeks of diet‐induced 12% weight loss followed by RCT of liraglutide 1.2 mg daily or placebo for 52 weeks	Denmark, n = 37 (all female) obese glucose‐tolerant, BMI 30 to 40, liraglutide, *n* = 18, placebo, *n* = 19	46 (liraglutide) and 45 (placebo)	Liraglutide increased bone formation marker P1NP by 16%, no change in CTX, and prevented bone loss after weight loss
Nissen et al.	2019	Examine acute effect of s.c. injections of intact GLP‐1 (7‐36)amide, as well as metabolite GLP‐1 (9‐36)amide, and degradation‐resistant GLP‐1RA, exenatide, on bone remodeling	Human, RCT, crossover, four study days (GLP‐1 (7‐36)amide, GLP‐1 (9‐36)amide, exenatide (exendin‐4), and saline)	Denmark, *n* = 8 (3 female/5 male), BMI 22	24	GLP‐1 (7‐36)amide and exenatide decreased CTX
Johansen et al.	2021	Evaluate efficacy of exenatide added to insulin in T1D on BMD and bone turnover markers	Human, RCT, exenatide 19 ug s.c. three times daily, 1 h before meal for 26 weeks	Denmark, *n* = 108 T1D, exenatide *n* = 52 (39 male/13 female), placebo *n* = 53 (37 male/16 female)	50	No change in BMD, CTX, or P1NP despite 4.4‐kg weight loss
Eriksson et al.	2018	Evaluate effect of exenatide on bone turnover and BMD in chronic, obese, antipsychotic‐treated subjects with schizophrenia	Human, RCT, exenatide 2 mg s.c. once weekly for 3 months	Denmark, *n* = 45 (female *n* = 24/male *n* = 21), exenatide *n* = 23, placebo *n* = 22	35.8	No significant effect on P1NP, CTX, or BMD; however, trend increase in BMD and decrease in P1NP
Iepsen et al.	2020	Evaluate impact of MC4R mutation bone mass and metabolism and impact on liraglutide on subjects	Human, liraglutide 3.0 mg once daily for 16 weeks	Denmark, obese subjects with MC4R mutations *n* = 17 and matched controls n = 35	33 (MC4R) and 42 (control)	6% weight loss in both groups but no differences in markers of bone turnover (CTX, osteocalcin, and P1NP); higher BMD increase in MC4R mutation when not corrected for body mass
Maagensen et al.	2020	Evaluate effect of liraglutide on bone turnover in patients with prediabetes and schizophrenia treated with olanzapine or clozapine	Human, RCT, liraglutide 0.6 to 1.8 mg daily for 16 weeks	Denmark, *n* = 103 with schizophrenia, prediabetes, BMI > 27, and treated with clozapine/olanzapine; liraglutide *n* = 35 (male *n* = 24), placebo *n* = 35 (male *n* = 54)	44 (liraglutide) and 42 (placebo)	Body weight loss and improvement of glucose tolerance but no change in P1NP or CTX
Animal and cell studies						
Nissen et al.	2019	Examine acute effect of GLP‐1 (7‐36)amide, GLP‐1 (9‐36)amide, and degradation‐resistant GLP‐1RA, exenatide, on COS‐7 cell culture	Cell study, COS‐7	N/A	N/A	All three GLP‐1 ligands acted as agonists of GLP‐1 receptor; GLP‐1 (7‐36) amide and exenatide acted as full agonists for cAMP formation, whereas GLP‐1(9‐36)amide acted as low‐potency partial agonist
Li et al.	2020	Examine effects of liraglutide on murine bone marrow‐derived macrophages and preosteoclasts	Cell study (liraglutide, receptor knock‐down, PCR, and western blot)	N/A	N/A	Identification of GLP‐1 R on murine macrophages and preosteoclasts; liraglutide inhibits osteoclast formation and bone resorption by inhibiting NF‐kB and MAPK pathways
Wu et al.	2017	Examine effect of liraglutide on murine preosteoblasts.	Cell study (liraglutien, PCR, and western blot)	N/A	N/A	Identified GLP‐1 R in murine pre‐osteoblasts; liraglutide promotes osteoblast proliferation by activating PI3K/AKT, ERK1/2, cAMP/PKA/β‐cat‐Ser675 signaling
Pereira et al.	2015	Examine effects of liraglutide versus exenatide versus saline on bone cells from mice; cell culturing, PCR, and serum analyses	Cell and animal; ovariectomized mice, GLP‐1 RA administration, histomorphometry analysis, and calcitonin and sclerostin; GLP‐1 receptor mRNA and protein expression	N/A	N/A	GLP‐1 RAs were identified in bone marrow cells, primary osteoclasts, osteoblasts, and osteocytes; exenatide increased osteoclast number and S‐calcitonin and decreased sclerostin levels
Zhai et al.	2023	Analyze GLP‐1 R during differentiation of human dental pulp stem cells into osteoblasts and effect of liraglutide in zebrafish bone formation	Cell culture of human dental pulp stem cells; PCR analysis, western blot, and ELISA; zebrafish scale	N/A	N/A	In stem cells: GLP‐1 R was identified during osteoblast differentiation, and liraglutide increased osteoblast differentiation by Runx2 signaling and calcium deposition; in zebrafish: GLP‐1 R activation induced osteoblast activity
Cheng et al.	2022	Examine effect of liraglutide versus insulin versus DPP4‐i (saxagliptin) on diabetic obese rats during weight loss	Cell and animal; rats, OGTT, micro‐CT, AGEs, mouse osteoblasts, PCR, western blot	N/A	N/A	Weight loss in untreated and liraglutide group, no weight loss in insulin and saxagliptin groups; liraglutide increased osteocalcin and ALP activity and decreased CTX with unchanged P1NP during weight loss; GLP‐1 attenuated Aminoglycoside‐induced inhibition of osteogenesis
Zhou et al.	2021	Examine antiosteoporotic effect of GLP‐1 RA on osteoporotic mice by comparing DBM, exenatide, and estradiol	Ovariectomized mice, intraperitoneal drug administration, ELISA, PCR, histology	N/A	N/A	BMD improved trabecular bone mass and bone strength, decreased CTX, and elevated P1NP; BMD increased gene expression of Runx2, ALP, Col1, and OC
Shen et al	2018	Investigate effect of exenatide in LPS‐induced osteoclast formation and bone resorption	Cell and animal; mice, drug injection, osteoclast number, PCR, μCT, serum	N/A	N/A	No in vitro effect of exenatide on RANKL‐induced osteoclast formation or TNF‐a‐induced osteoclast formation; in vivo, exenatide lowered osteoclast number, bone resorption, and CTX and inhibited RANKl and TNF‐a mRNA expression
Wang et al.	2017	Examine effect of exenatide on bone and bone marrow‐derived macrophage surface migration	Cell and animal; ovariectomized mice, drug injection, μCT, ELISA, PCR, western blot, SiRNA, migration	N/A	N/A	In vivo, exenatide increased bone formation and improved trabecular bone structure; in vitro, exenatide increased bone marrow‐derived macrophages on bone surface
Ma et al.	2013	Examine effect of exenatide on bone remodeling	Cell and animal; ovariectomized rats, drug injection, DXA, μCT, ELISA, PCR, western blot, bone marrow stem cells	N/A	N/A	Exenatide increased BMC, BMD, trabecular bone structure, bone strength, and bone formation (P1NP) and inhibited bone resorption (CTX)
Meng et al.	2016	Investigate effect of exenatide on bone marrow stem cell differentiation and bone formation	Cell and animal; rats, drug injection, mechanical unloading, bone marrow stem cells from rat femur and tibia; ELISA, μCT, histology, western blot	N/A	N/A	GLP‐1 R not expressed in primary osteoblasts but in insulinoma cells and bone marrow stem cells; exenatide increased trabecular bone mass, bone strength, osteoblast number, bone formation markers (OC and P1NP) and suppressed adipocyte differentiation in bone marrow stem cells; exenatide treatment increased B‐catenin (Wnt pathway), Runx2, and osterix
Pereira et al.	2017	Examine exenatide's effect on bone quality and strength	Cell and animal; diabetic leptin receptor deficient mice, drug injection, μCT, histomorphometry, BMD, osteoblast cells, limb perfusion	N/A	N/A	In vitro: only effect on bone formation (in osteoblasts) during high blood glucose concentrations; exenatide decreased blood glucose in diabetic mice and lowered body mass in obese but not in lean mice; tibial vasodilation in exanatide‐treated mice; exanatide improved trabecular bone and increased osteoblast activity and number in obese diabetic mice; no differences observed in number of TRACP‐positive osteoclastic surfaces
Wang et al.	2020	Examine effect of a GLP‐1 polymer on bone regeneration	Cell and animal; mice, bone defect, drug injection, murine bone marrow‐derived macrophages and stem cells, PCR, western blot	N/A	N/A	GLP‐1 polymer induced higher osteogenesis and suppressed adipogenesis; osteocalcin, osterix, and Runx2 increased and PPARγ decreased

DPP‐4is and bone turnover						
Human studies						
Buck et al.	2012	Assess whether DPP‐4i affects markers of bone resorption and calcium homeostasis	Human study: single‐center, double‐blind, RCT	1‐year treatment with DPP‐4i vildagliptin (100 mg, once daily; *n* = 29) or placebo (*n* = 30)		Serum CTX concentrations compared with pretreatment levels were not changed (between‐group ratio 1.15 ± 0.17; *p* = 0.320); fasting serum ALP, calcium, and phosphate were also unaffected
Animal and cell studies						
Xiang et al.	2020	Examine angiogenesis with sitagliptin treatment in diabetes‐induced poor osteointegration	Cell and animal study: in vitro and in vivo rat study	N/A	N/A	Study on diabetes induced by high‐fat diet and streptozotocin reported that cell growth was disturbed while both abnormal macrophage polarization and endothelial impairment in diabetes were significantly alleviated by sitagliptin
Nirwan et al.	2022	Investigate effects of linagliptin and metformin alone and in combination in treating diabetic osteoporosis in high‐fat‐fed mice	Animal study: in vitro study of mice	N/A	N/A	Treatment with linagliptin alone and in combination with metformin significantly reverted impaired bone architecture and BMD and positively modulated bone turnover biomarkers
Eom et al.	2016	Investigate effects of DPP‐4i on TZD induced bone loss in T2D animal model	Animal study: T2D rat model	N/A	N/A	Osteocalcin levels decreased and TRACP 5b increased in pioglitazone group
Glorie et al.	2014	Evaluate effect of DPP‐4i sitagliptin on bone in normal and streptozotocin‐induced diabetic rats	Animal study: T2D Wistar rats versus control rats	N/A	N/A	Serum levels of CTX‐I were significantly lower in sitagliptin‐treated diabetic animals compared with untreated diabetic animals

Abbreviation: AGEs = Advaned glycated endpoints; ALP = alkaline phosphatase; BMD = bone mineral density; BMI = body mass index; cAMP = Cyclic adenosinmonofosfat; CT = computed tomography; CTX = C‐terminal telopeptide of collagen; DMB = A quinoxaline‐based compound investigated as a GLP‐1 RA; LPS = lipopolysaccharide; MC4R = melanocortin‐4 receptor; NF‐κB = Nuclear factor kappa‐light‐chain‐enhancer of acivated B‐cells; OGTT = oral glucose tolerance test; P1NP = procollagen I N‐terminal propeptide; PCR = Poly chain Reaction; RANKL = receptor activator of nuclear factor‐κB ligand; RCT = randomized controlled trial; SU = sulfonylurea; T2D = type 2 diabetes; TNF = Tumor Necrosis Factor; TZD = thiazolidinediones.

**Table 3:** Overview of Studies of Bone Structure with Glucagon‐like Peptide 1 Receptor Agonists (GLP‐1 RAs) and Dipeptidyl Peptidase 4 Inhibitors (DPP‐4is)

Authors	Year	Purpose	Research design	Population (country, *n*)	Age (years)	Main outcomes
GLP‐1 RAs and bone structure						
Human studies						
Lepsen et al.	2020	Evaluate impact of MC4R mutation bone mass and metabolism and impact of liraglutide on subjects	Human, liraglutide 3.0 mg once daily for 16 weeks	Denmark, obese subjects with MC4R mutations, *n* = 17, and matched controls, *n* = 35	33 (MC4R) and 42 (control)	6% weight loss in both groups; liraglutide induced higher BMD in MC4R mutation when not corrected for body mass
Cai et al.	2021	Effect of exenatide, dulaglutide, and insulin on BMD in T2DM	Human; single‐blinded; exenatide, dulaglutide, insulin	China. Patients with T2DM *n* = 65. Exenatide *n* = 19 (male/female 9/10), dulaglutide *n* = 19 (male/female 11/8), insulin glargine *n* = 10 (male/female 7/3), placebo *n* = 17 /male/female 9/8)	> 40 (63, 57, 64, 62)	Total hip BMD increased with exenatide treatment; femoral neck BMD decreased in dulaglutide (but less than placebo); lumbar BMD increased in insulin group; compared to placebo, exenatide and insulin increased BMD of femoral neck and total hip
Hygum et al.	2019	Investigate effect of liraglutide on bone turnover in subjects with T2D	Human, RCT, liraglutide 1.8 mg daily or placebo for 26 weeks	Denmark, T2D patients, *n* = 60 liraglutide, *n* = 30 (14 female/16 male) and placebo, n = 30 placebo (16 female/14 male)	62 (liraglutide) and 64 (placebo)	Hip BMD decreased in placebo group and was unchanged in liraglutide group
Huang et al.	2023	Determine how switching from DPP‐4i to GLP‐1RA influenced BMD in diabetic patients	Human, retrospective cohort study.	Taiwan, T2D treated with DPP‐4i and with osteopenia/osteoporosis, switchers to GLP‐1 RA, *n* = 132, matched to 133 nonswitchers	62 (switchers) and 63 (control)	Switching to GLP‐1 RA decreased lumbar spine BMD more than continuing a DPP‐4i (−0.028 g/cm^2^ versus −0.019 g/cm^2^, *p* = 0.041, adjusted for BMI)
Johansen et al.	2021	Evaluate efficacy of exenatide added to insulin in T1D on BMD	Human, RCT, exenatide 19 ug s.c. three times daily, 1 h before meal for 26 weeks	Denmark, *n* = 108 type 1 diabetes, exenatide *n* = 52 (39 male/13 female), placebo *n* = 53 (37 male/16 female)	50	No change in BMD
Eriksson et al.	2018	Evaluate effect of exenatide on BMD in chronic, obese, antipsychotic‐treated subjects with schizophrenia	Human, RCT, exenatide 2 mg s.c. once weekly for 3 months	Denmark, *n* = 45 (female *n* = 24/male *n* = 21), exenatide *n* = 23, placebo *n* = 22	35.8	No significant effect on BMD; however, trend increase in BMD and decrease in P1NP
Iepsen et al.	2015	Investigate role of liraglutide in bone formation and weight loss‐induced bone mass reduction	Human, 8 weeks of diet‐induced 12% weight loss followed by RCT of liraglutide 1.2 mg daily or placebo for 52 weeks	Denmark, *n* = 37 (all female) obese glucose‐tolerant, BMI: 30 to 40, liraglutide, *n* = 18, placebo, *n* = 19.	46 (liraglutide) and 45 (placebo)	Liraglutide prevented bone loss after weight loss

Animal and cell studies						
Yamada et al.	2008	Investigate role of GLP‐1 in regulation of bone metabolism using GLP‐1 receptor knockout	Animal; GLP‐1 R knockout mice and exenatide treatment, μCT	N/A	N/A	Lower total and cortical BMD, no difference in trabecular bone; exenatide increased calcitonin gene expression
Wang et al.	2020	Examine effect of a GLP‐1 polymer on bone regeneration	Mice, bone defect, drug injection, μCT	N/A	N/A	GLP‐1 polymer induced higher trabecular bone scores
Abdi et al.	2023	Compare effects of GLP‐1 RA and DPP‐4 inhibitor on bone biomechanical properties	Animal: diabetic rats, exenatide treatment	N/A	N/A	Reduced osteoclast function; restored bone elasticity but deteriorated bone strength; exanatide more effective than DPP‐4i
Sun et al.	2015	Examine liraglutide's effect on rat bone	Animal: diabetic rats	N/A	N/A	Improved trabecular and cortical bone measures
Pereira et al.	2015	Examine effects of liraglutide versus exenatide versus saline on bone	Animal. Ovariectomized mice, GLP‐1 RA administration, μCT	N/A	N/A	GGLP‐1 RAs increased trabecular but not cortical bone indices
Cheng et al.	2022	Examine effect of liraglutide versus insulin versus DPP4‐i (saxagliptin) on diabetic obese rats during weight loss	Animal: rats, μCT	N/A	N/A	Weight loss in untreated and liraglutide group, no weight loss in insulin and saxagliptin groups; trabecular bone structure and bone strength improved in liraglutide‐ and insulin‐treated rats
Zhou et al.	2021	Examine antiosteoporotic effect of DMB (a GLP‐1 RA) on osteoporotic mice by comparing DBM, exenatide, and estradiol	Ovariectomized mice, intraperitoneal drug administration, μCT	N/A	N/A	DMB improved trabecular bone mass and bone strength
Deng et al.	2019	Examine exantide effect on mouse bone repair	Animal: mice, μCT	N/A	N/A	Exenatide increased recovery of damaged bone tissue
Parthsarathy et al	2017	Examine exenatide effect on mouse bone	Animal: mice, DXA	N/A	N/A	Exenatide increased BMC, no effect on BMD
Yu et al.	2021	Examine liraglutide's effect on mouse bone	Animal: diabetic (T1D) female mice, ± liraglutide and μCT	N/A	N/A	Liraglutide treatment improved trabecular spacing and size, as well as trabecular and cortical BMD in tibia and partial recovery of trabecular microarchitecture; no effect on bone turnover markers
Wen et al.	2018	Examine liraglutide's effect on diabetic rat bone	Animal: diabetic and ovariectomized rats, liraglutide, microscopic evaluation	N/A	N/A	Liraglutide treatment maintained and attenuated BMD after diabetes induction and ovary removal; further, liraglutide decreased CTX and RANKL/OPG ratio
Lu et al.	2015	Examine liraglutide's effect on bone of ovariectomized rats	Animal: ovariectomized rats without diabetes, liraglutide, μCT			Liraglutide improved trabecular volume, thickness, and number, increased BMD, and reduced trabecular spacing in femur
Pereira et al.	2017	Examine exenatide's effect on bone quality and strength	Animal: diabetic leptin receptor‐deficient mice, drug injection, μCT	N/A	N/A	Tibial vasodilation in exanatide‐treated mice; exanatide improved trabecular bone and increased osteoblast activity and number in obese diabetic mice
Mansur	2019	Evaluate impact of high‐fat‐diet‐induced diabetes and therapy using clinically approved GLP‐1 RA exenatide on tissue bone mechanical properties and compositional parameters	Mice, ad libitum high‐fat diet + diabetes	N/A	N/A	Exenatide did not ameliorate BMD at any site but affected bone mechanical properties positively, e.g., higher strain and load
Mieczkowska et al.	2020	Compare head to head the effects of dapagliflozin and liraglutide on bone strength and bone material properties in a preclinical model of diabetes‐obesity	Mice, dapagliflozin versus liraglutide on bone strength and material properties on diabetic mice			No effect on bone strength or bone microarchitecture; different effects of SGLT2‐i and GLP‐1 RA on bone properties that were not only mediated by lowering blood glucose; liraglutide administration resulted in lower crystal size index, higher mature collagen crosslinks, higher collagen maturity, and lower collagen glycation

DPP‐4is and bone structure						
Human studies						
Qui et al.	2020	Investigate relationship between plasma DPP4 activity and osteoporosis/osteopenia and fracture risk in newly diagnosed T2D	Human study: cross‐sectional study	*N* = 147 people with newly diagnosed T2D	Between 53 and 57 years of age	Elevated plasma DPP activities were associated with higher proportion of osteoporosis/osteopenia in subjects with newly diagnosed T2D
Lee et al.	2021	Investigate effect of DPP‐4is on bone health using TBS in patients with T2D	Human study: single‐center, retrospective case–control study	*N* = 200 patients with T2D	68 ± 10 years	BMD increased in both DPP‐4 exposed and unexposed, with no differences between groups

Animal and cell studies						
Bautista et al.	2019	Investigate effects of sitagliptin, a dipeptidyl peptidase 4 inhibitor used to treat T2D, on bone tissue and on implant osseointegration in diabetic rats	Animal study: T2D rats versus normoglycemic rats	N/A	N/A	Sitagliptin administration did not reverse negative effects of T2D on bone
Ishida et al.	2018	Investigate in vivo effects of DPP‐4 inhibition on LPS‐induced osteoclast formation and bone resorption, as well as in vitro effects of DPP‐4 inhibition on RANKL‐induced osteoclast genesis and TNF‐α‐induced osteoclast genesis	Animal study of mice	N/A	N/A	A lower osteoclast number and decreased bone resorption in mice that underwent LPS and DPP‐4 inhibitor co‐administration compared with LPS administration alone
Eom et al.	2016	Investigate effects of a DPP‐4i on TZD‐induced bone loss in T2DM animal model	Animal study of T2D mice	N/A	N/A	Vildagliptin treatment significantly increased BMD and trabecular bone volume; combination therapy restored BMD, trabecular bone volume, and trabecular bone thickness
Glorie et al.	2014	Evaluate effect of DPP‐4i sitagliptin on bone in normal and streptozotocin‐induced diabetic rats	Animal study: T2D Wistar rats versus control rats	N/A	N/A	Sitagliptin prevented cortical bone growth stagnation in diabetic rats assessed by μCT, resulting in stronger femora during three‐point bending

Abbreviation: BMD = bone mineral density; CT = computed tomography; CTX = C‐terminal telopeptide of collagen; DMB = A quinoxaline‐based compound investigated as a GLP‐1 RA; LPS = lipopolysaccharide; MC4R = melanocortin‐4 receptor; RCT = randomized controlled trial; T2D = Type 2 diabetes.

**Table 4:** Overview of Cell Studies with Glucagon‐like Peptide 1 Receptor Agonists (GLP‐1 RAs) and Dipeptidyl Peptidase 4 Inhibitors (DPP‐4is)

Authors	Year	Purpose	Research design	Main outcomes
GLP‐1 RAs and Cell studies				
Li et al.	2020	Investigate effects of liraglutide, a GLP‐1 RA, on murine bone marrow‐derived macrophage (BMM) and RAW264.7 preosteoclast differentiation and explore potential cellular basis of its action	In vitro	Study confirmed presence of GLP‐1 receptor (GLP‐1R) on BMMs and RAW264.7 cells and demonstrated that GLP‐1R might be important for osteoclastogenesis by increasing expression of osteoclastogenic biomarkers after GLP‐1R knockdown; in addition, we found that liraglutide treatment of both BMMs and RAW264.7 cells could inhibit osteoclast formation and bone resorption
Wu et al.	2017	Investigate effects of liraglutide, a GLP‐1 RA, on murine MC3T3‐E1 preosteoblast proliferation and differentiation and explore potential cellular basis	In vitro	Study confirmed presence of GLP‐1R in MC3T3‐E1 and demonstrated that liraglutide promoted osteoblast proliferation at intermediate concentration (100 nM) and time (48 h), upregulated expression of osteoblastogenic biomarkers at various stages, and stimulated osteoblastic mineralization; liraglutide also elevated intracellular cAMP level and phosphorylation of AKT, ERK, and β‐catenin simultaneously with increased nuclear β‐catenin content and transcriptional activity
Zhai et al.	2023	Investigate role of dental‐derived stem cells (hDPSCs) and their association with GLP‐1 R	In vitro	GLP‐1 RA expression was found to be upregulated during osteoblast differentiation; GLP‐1 RA liraglutide peptide treatment increased osteoblast differentiation in hDPSCs by increasing calcium deposition, ALP activity, and osteoblast marker genes, Runx2, type 1 col, osteonectin, and osteocalcin

DPP‐4is and cell studies				
Xiang et al	2020	Examine role of angiogenesis with sitagliptin treatment in diabetes‐induced poor osteointegration of titanium implants and underlying mechanisms	In vitro, human umbilical vein endothelial cells (HUVECs) incubated on titanium (Ti) surface were subjected to (1) normal milieu (NM); (2) diabetic milieu (DM); (3) DM + sitagliptin; (4) NM + macrophage; (5) DM + macrophage; or (6) DM + macrophage + sitagliptin	In vitro, when cells were incubated alone, DM caused M1 polarization of macrophage, evidenced by increased iNOS and decreased CD206 expressions, and obvious dysfunctions of HUVECs; DM‐induced injury of endothelial cells was significantly worsened when the two cells were co‐cultured; addition of sitagliptin markedly reversed changes of macrophage but not of HUVECs in DM when cells were cultured alone; when cells were co‐cultured, however, both abnormal macrophage polarization and endothelial impairment in DM was significantly alleviated by sitagliptin

*Note*: The MAPK/ERK pathway (also known as the Ras‐Raf‐MEK‐ERK pathway) is a chain of proteins in the cell that communicates a signal from a receptor on the surface of the cell to the DNA in the nucleus of the cell. cAMP = cyclic adenosinmonophosfat; iNOS = Nitric oxide synthase.

### GLP‐1 RAs

#### Fractures

In general, a cohort study comparing GLP‐1 RAs and DDP‐4is showed no significant difference in incident major osteoporotic fractures.^(^ ^27^ ^)^ Using the same cohort, the authors reported no difference in major osteoporotic fracture risk between GLP‐1 RAs or SGLT2is (both in combination with metformin).^(^ ^28^ ^)^ Another cohort study using people aged ≥65 years from Medicare reported that initiation of SGLT‐2 inhibitors was not associated with an increased risk of fracture in older T2D adults compared with initiating a GLP‐1 RA.^(^ ^29^ ^)^ Compared with insulin, a network meta‐analysis using data from RCTs did not report any increase in fracture risk with GLP‐1 RAs,^(^ ^30^ ^)^ although a meta‐analysis using observational studies concluded that current GLP‐1 RA use was associated with a decreased risk of fracture.^(^ ^31^ ^)^ This finding was consistent with the results from two additional cohort studies conducted by the same group of authors.^(^ ^32^, ^33^ ^)^

An older meta‐analysis found no reduction in fracture risk by GLP‐1 RAs in T2D compared to other antidiabetic medications.^(^ ^34^ ^)^ Interestingly, another older meta‐analysis of RCTs reported that, compared with placebo and other antidiabetic drugs, liraglutide and lixisenatide were associated with a significant reduction in the risk of bone fractures, and the beneficial effects were dependent on the treatment duration, with effects only observed after more than 1 year.^(^ ^35^ ^)^ Yet another meta‐analysis also looked at subtypes of drugs from RCTs and reported that albiglutide (GLP‐1‐RA) decreased the risk of fracture (relative risk [RR] of 0.29 [95% CI: 0.04 to 0.93]) compared to placebo.^(^ ^36^ ^)^ None of the other subtypes of GLP‐1 RAs were significantly associated with fracture risk. Additionally, a newer meta‐analysis of RCTs reported that GLP‐1 RAs were associated with a decreased fracture risk compared to placebo or other antihyperglycemic drugs, and the reduction was highest for exenatide.^(^ ^37^ ^)^ Yet an older meta‐analysis of RCTs reported that liraglutide was associated with a reduced risk of incident fractures (odds ratio [OR] of 0.38 [95% CI: 0.17 to 0.87]), whereas exenatide was associated with an elevated risk (OR of 2.09 [95% CI: 1.03 to 4.21]).^(^ ^38^ ^)^

This section presents a collection of cohort studies, meta‐analyses, and network meta‐analyses that investigate the association between GLP‐1 RAs with the risk of fractures in people with T2D. In summary, the majority of the studies showed either no risk^(^ ^27^, ^28^, ^29^, ^30^ ^)^ or a decreased risk^(^ ^31^, ^32^, ^33^, ^35^, ^36^, ^37^, ^38^ ^)^ including certain subtypes like liraglutide,^(^ ^35^ ^)^ lixisenatide,^(^ ^35^ ^)^ albiglutide,^(^ ^36^ ^)^ and exenatide.^(^ ^37^ ^)^

#### Bone turnover and bone structure

##### Human studies

The section presents various clinical trials and studies that investigate the effects of GLP‐1 RAs in human studies, particularly liraglutide and exenatide, on bone metabolism, BMD, and bone turnover markers in people with diabetes and in overweight.

The effects of liraglutide on bone resorption and turnover was exploited in two trials. One RCT that included T2D people found that liraglutide treatment for 6 months did not affect bone resorption despite a significant weight loss compared to the control group without weight loss.^(^ ^39^ ^)^ These observations may suggest that liraglutide expresses antiresorptive effects. Moreover, a trial on healthy obese people exposed to liraglutide for 52 weeks after a 12% weight loss intervention reported increased P1NP and no change in CTX in the liraglutide group compared to the control group, which indicates a higher bone formation.^(^ ^40^ ^)^

The exenatide effects on bone metabolism in healthy T1D people were discussed in three studies. One human trial included healthy normal‐weight controls and showed suppression of CTX after exenatide injection.^(^ ^41^ ^)^ Another study in T1D people concluded that, despite an exenatide‐induced body weight reduction, no changes in bone metabolism were observed with exenatide added to insulin therapy after 26 weeks.^(^ ^42^ ^)^ A double‐blinded RCT investigated the effects of exenatide 2 mg once weekly (*n* = 23) or placebo (*n* = 22) on bone turnover markers in chronic, obese, antipsychotic‐treated people.^(^ ^43^ ^)^ They reported, in general, no significant effects on bone turnover markers (P1NP and CTX) after 3 months of treatment. However, they did observe a numerical reduction of both PINP and CTX in the exenatide group and a numerical increase in the placebo group.^(^ ^43^ ^)^ These findings suggest that weight‐induced bone turnover is mitigated by GLP‐1.

The effects of liraglutide on bone metabolism in healthy and obese. A clinical trial included people (without diabetes) with common obesity and people with one of the most common monogenic causes of obesity in humans, a mutation in the melanocortin‐4 receptor (MC4R).^(^ ^44^ ^)^ They observed a 6% weight loss in both groups but no differences in markers of bone turnover (CTX, osteocalcin and P1NP) after 16 weeks of liraglutide treatment (neither in groups of between groups). Although not directly related to diabetes, the use of liraglutide in people with prediabetes due to olanzapine‐ or clozapine‐treated schizophrenia did not change bone turnover markers.^(^ ^45^ ^)^ Atrial that involved obese people with and without an MC4R mutation, it was observed that the control group exhibited an increase in bone mass (measured by mineral apparent density) in response to liraglutide treatment. However, no such increase was observed in the MC4R group.^(^ ^44^ ^)^

To elucidate the effects of exenatide and dulaglutide on BMD in people with diabetes, a single‐blind study in overweight T2D people randomized to exenatide, dulaglutide, insulin glargine, or placebo reported a BMD increase of the total hip in the exenatide group. In the dulaglutide‐treated group, only the femoral neck BMD decreased, but the magnitude of the decrease was less than that observed in the placebo group; the BMD of the first to fourth lumbar vertebrae (L1–L4), femoral neck, and total hip decreased significantly in the placebo group, while in the insulin glargine group, the BMD of L2, L4, and L1‐4 increased.^(^ ^46^ ^)^ Compared with the placebo group, the BMD of the femoral neck and total hip increased significantly in the exenatide group and the insulin glargine group. Compared with the exenatide group, the BMD of L4 in the insulin glargine group increased as well.^(^ ^47^ ^)^ In the RCT on T2D people randomized to liraglutide or placebo, mentioned earlier, liraglutide treatment prevented a hip BMD decrease (despite a weight loss), as was otherwise observed in the control group.^(^ ^39^ ^)^ The effect was observed as early as 13 weeks after initiation. No differences were observed for lumbar spine BMD or in the evaluation of bone microstructure by HR‐pQCT.^(^ ^39^ ^)^

GLP‐1 RA treatment on BMD in T2D people was first evaluated in a retrospective cohort study that included people with diabetes and compromised bone quality (*T*‐score < −1), the effects of switching from a DPP‐4i to a GLP‐1 RA were examined. Although an improvement of glycemic control and a larger weight loss was observed, the GLP‐1 RA group experienced a more significant decrease in the lumbar spine BMD than the group continuing DPP‐4i treatment (−0.028 g/cm^2^ versus −0.019 g/cm^2^, *p* = 0.041, adjusted for body mass index [BMI]).^(^ ^48^ ^)^ However, in the study examining bone turnover markers, a 6‐month exposure to exenatide in people with T1D did not result in any changes in BMD, as assessed through measurements of the whole body, hip, lumbar spine, and forearm.^(^ ^42^ ^)^ Furthermore, in a trial involving obese people without diabetes who were on antipsychotic treatment, a 3‐month exposure to exenatide did not lead to any changes in BMD in the lumbar spine, femoral neck, or total hip.^(^ ^43^ ^)^

Lastly, the effect of switching from DPP‐4i to GLP‐1 RA on BMD was seen in a trial conducted on healthy obese people, the effects of liraglutide over a period of 52 weeks were investigated. It was found that during weight maintenance, the control group experienced a decrease in total, pelvic, and arm‐leg BMC. However, no changes were observed in these measurements in the liraglutide treatment group.^(^ ^40^ ^)^

Overall, the studies suggest that GLP‐1 RAs, particularly liraglutide^(^ ^39^, ^40^, ^44^, ^45^ ^)^ and exenatide,^(^ ^41^, ^42^, ^43^, ^46^, ^47^ ^)^ may have varying effects on bone metabolism and BMD in different populations, including T2D people and obese people, although the effect of the drugs is generally neutral. The impact on bone health appears to be influenced by factors such as weight loss, genetic mutations, and the presence of diabetes.

##### Animal studies

This section provides an overview of several animal studies investigating the effects of GLP‐1 RAs, particularly liraglutide and exenatide, on bone health, BMD, bone turnover markers, and bone strength.

First, the effects of liraglutide on bone remodeling and bone loss was evaluated. An animal study on rats showed that liraglutide improved bone remodeling parameters by increasing the bone formation marker osteocalcin and the activity of alkaline phosphatase (ALP) while decreasing CTX (a bone resorption marker) with no change in P1NP (another bone formation marker).^(^ ^49^ ^)^ In ovariectomized mice, both liraglutide and a quinoxaline‐based compound prevented bone loss, as evidenced by increased P1NP and decreased CTX levels, indicating enhanced bone formation and reduced bone resorption. However, these effects were observed only with exenatide treatment, not with liraglutide.^(^ ^50^, ^51^ ^)^ A study evaluated the effects on bone mediated by the SGLT2 inhibitor dapagliflozin versus liraglutide and reported a different but positive impact on bone microarchitecture and material properties by both drugs that was not explained by the lowering of blood glucose.^(^ ^52^ ^)^ They reported that liraglutide administration resulted in higher mature collagen crosslinks and lower collagen glycation as well as restoring a normal postprocessing of collagen molecules. These findings may indicate the ability of GLP‐1 RA to restore the immature divalent collagen crosslinks seen in diabetes.

Then the effects of exenatide on bone resorption and bone healing was assessed. A study using exenatide on mice reported a lowering of bone resorption measured by CTX and the receptor activator of nuclear factor‐κB ligand (RANKL); the latter acts as an essential part of osteoclast formation and activation.^(^ ^53^ ^)^ The study further investigated any potential mechanism and found that GLP‐1 RAs may affect the inhibition of the lipopolysaccharide (LPS)‐induced TNF‐*α* expression of macrophages more than a direct effect on osteoclast precursors or RANKL expression. LPS promotes inflammation and inflammatory bone loss and induces the production of proinflammatory cytokines, e.g., TNF‐*α*, and TNF‐*α* enhances osteoclast formation and RANKL expression on stromal cells.^(^ ^54^ ^)^ Thus, these results suggest an inhibition of LPS‐induced osteoclast formation by GLP‐1 RA.^(^ ^53^ ^)^ Other studies have also provided additional support for these findings.^(^ ^55^, ^56^, ^57^ ^)^

The effects of GLP‐1 RAs on bone quality in various mice models was assessed in several studies. One study used a leptin receptor‐deficient mice model with obesity and severe T2D to assess changes in bone quality after 4 weeks of exenatide administration.^(^ ^58^ ^)^ They reported an increased bone formation rate in leptin‐deficient mice but no effect on lean mice also exposed to exenatide treatment. They did not observe an effect on bone resorption. Then, a study on mice with a femoral defect treated with a GLP‐1 polymer found an increased number of osteoblasts and decreased number of osteoclasts with corresponding effects on the expression of formation and resorption markers, suggesting that the GLP‐1 polymer enhances bone formation.^(^ ^59^ ^)^ A study from 2008 investigated GLP‐1 RA knockout mice and reported lower total and cortical BMD evaluated by a computed tomography (CT)‐based analysis with no difference in the trabecular evaluation.^(^ ^60^ ^)^ The study on mice with a femoral defect also analyzed by micro‐CT (μCT) and observed higher trabecular number, trabecular thickness, and bone volume to total volume (BV/TV) ratio in the mice treated with a polymer of GLP‐1.^(^ ^59^ ^)^ These findings suggest a promoted bone formation and a potential increasing rate of bone healing facilitated by GLP‐1.

GLP‐1 RAs' effects on BMD and bone strength in several mice models. In a short‐term animal study lasting 5 weeks, it was found that the antidiabetic treatment using exenatide as a GLP‐1 RA and sitagliptin as a DPP‐4i restored bone elasticity in comparison to untreated diabetic rats. However, the treatment resulted in a deterioration of bone strength.^(^ ^61^ ^)^ Both the untreated diabetic rats and the treated rats showed significantly higher bone bending stress, but this was improved by the treatment, with exenatide demonstrating a slightly more pronounced effect than sitagliptin.^(^ ^61^ ^)^ Another study involving diabetic rats reported improvements in trabecular and cortical bone measures after 4 weeks of liraglutide treatment.^(^ ^62^ ^)^ In a study conducted on ovariectomized mice, both liraglutide and exenatide were found to enhance bone mass, as indicated by increased trabecular bone indices measured using μCT.^(^ ^50^ ^)^ Notably, no changes were observed in cortical bone measurements. Another animal study demonstrated that liraglutide prevented the deterioration of trabecular microarchitecture and improved bone strength.^(^ ^49^ ^)^ Additionally, a different animal study showed that a GLP‐1 RA improved bone loss primarily through the induction of bone formation.^(^ ^51^ ^)^ Studies on nondiabetic mice also revealed that exenatide increased the transcription of osteogenic differentiation‐related genes, promoted osteogenic differentiation and bone repair, and improved BMC.^(^ ^63^, ^64^ ^)^

In a short‐duration study conducted on animals with T1D lasting 8 weeks, liraglutide treatment, either alone or in combination with insulin, was found to restore decreased BMD and partially correct compromised trabecular microarchitectures compared to control animals without T1D without any changes in bone turnover markers.^(^ ^65^ ^)^ A trial involving ovariectomized rats with streptozotocin‐induced diabetes reported a reduction in femoral BMD and the destruction of bone microarchitecture, which was alleviated by liraglutide treatment along with decreased RANKL/OPG ratio.^(^ ^46^ ^)^ Evaluation based on counted numbers of osteoblasts and osteoclasts using H&E‐ and TRACP‐stained sections was used.^(^ ^46^ ^)^ Another trial conducted on ovariectomized rats without diabetes found that liraglutide improved trabecular volume, thickness, and number, increased BMD, and reduced trabecular spacing in the femurs, as determined by μCT analysis. Similar results were observed in the lumbar vertebrae.^(^ ^66^ ^)^ In a study involving leptin receptor‐deficient mice exposed to exenatide, improved trabecular bone structure was observed. Consistent with the aforementioned studies, no differences were found in the cortical bone parameters following exenatide treatment.^(^ ^58^ ^)^ Lastly, biomechanical bone strength was evaluated in six studies. Two studies found an improvement of bone strength by GLP‐1 RAs^(^ ^51^, ^57^ ^)^ one study found that bone elasticity was restored but bone strength deteriorated,^(^ ^61^ ^)^ and two studies found no effect on bone strength.^(^ ^52^, ^58^ ^)^ Though results were inconsistent, most animal studies suggested an improved or no effect on bone strength after GLP‐1 RA therapy.

Overall, animal studies suggest that GLP‐1 RAs, particularly exenatide^(^ ^53^, ^54^, ^55^, ^56^, ^57^, ^61^, ^63^, ^64^ ^)^ and liraglutide,^(^ ^46^, ^49^, ^50^, ^51^, ^65^ ^)^ may have beneficial effects on bone health by promoting bone formation, improving bone microarchitecture, and potentially preventing bone loss. However, the effects on bone strength are less consistent and may depend on the specific GLP‐1 RA used and the study conditions.

#### Cell studies

The section discusses several cell culture studies conducted on rodents, including bone marrow mesenchymal stem cells, bone marrow‐derived macrophages, fibroblasts, preosteoclasts, and osteoblasts, to investigate the presence and effects of GLP‐1 RAs on bone remodeling processes.

As for the presence and effects of GLP‐1RAs on bone cells, several studies on cell cultures from rodents, including bone marrow mesenchymal stem cells, bone marrow‐derived macrophages, fibroblasts, and preosteoclasts, demonstrated that the presence of GLP‐1 receptors and GLP‐1 RAs (mainly exenatide) could potentially inhibit bone resorption and promote osteogenesis and osteoblast proliferation.^(^ ^50^, ^57^, ^67^ ^)^ These findings suggest that GLP‐1 binds to and modulates cells involved in bone remodeling in favor of formation by increasing osteoclast number and serum CTX. However, mixed evidence exists on the effect of GLP‐1 receptor presence on osteoblasts, with one study reporting that GLP‐1 receptors were not identified on primary osteoblasts cultured from rat bone marrow stem cells but another study reporting the presence of GLP‐1 receptors on osteoblasts cultured from murine preosteoblasts.^(^ ^68^ ^)^ However, yet another study reported that exenatide only influenced bone nodule formation of cultured primary murine osteoblasts during high glucose concentrations.^(^ ^58^ ^)^

Another study exploited human dental pulp‐derived stem cells and observed increased osteoblast differentiation during liraglutide treatment, high activity of bone ALP, and increased expression of osteoblast marker genes, e.g., Runx2, type 1 collagen, osteonectin, and osteocalcin.^(^ ^69^ ^)^

Overall, the cell culture studies suggest that GLP‐1 RAs are present on various bone cells, and GLP‐1 RAs, especially exenatide and liraglutide, have the potential to promote bone formation and inhibit bone resorption. However, the presence of GLP‐1 RAs on osteoblasts remains a subject of debate due to some contradictory findings. These studies provide valuable insights into the potential mechanisms through which GLP‐1 RAs might impact bone metabolism, supporting the notion that these drugs could have beneficial effects on bone health.

### DPP‐4is

#### Fractures

The section presents a collection of cohort studies, meta‐analyses, and network meta‐analyses that investigate the association between the use of DPP‐4is and the risk of fractures in people with diabetes.

Several meta‐analyses have been conducted. One meta‐analysis^(^ ^70^ ^)^ reported that as a group, incretins were not associated with fracture risk (OR of 0.97 [95% CI: 0.88 to 1.08]) and another of RCTs^(^ ^71^ ^)^ concurred, specifically on DPP‐4is (RR of 0.83 [95% CI: 0.60 to 1.14]).

Regarding specific subtypes of incretins, a meta‐analysis suggested that the risk of fracture for alogliptin was decreased compared to placebo (OR of 0.51 [95% CI: 0.29 to 0.88]).^(^ ^72^ ^)^ Aloglitpin also reportedly reduces the fracture risk compared with linagliptin (OR of 0.45 [95% CI: 0.20 to 0.99]) and saxagliptin (OR of 0.46 [95% CI: 0.25 to 0.84]); the risk was higher with saxagliptin versus sitagliptin (OR of 1.90 [95% CI: 1.04 to 3.47]) and sulfonylureas (OR of 1.98 [95% CI: 1.06 to 3.71]). In a direct pairwise meta‐analysis, alogliptin was associated with a nonsignificant tendency to fracture risk reduction compared with placebo (OR of 0.54 [95% CI: 0.29 to 1.01]).^(^ ^72^ ^)^ In addition, from the first meta‐analysis, a subgroup analysis revealed that sitagliptin 100 mg daily (OR of 0.495 [95% CI: 0.30 to 0.80]) was associated with a reduced risk of fractures.^(^ ^70^ ^)^ Interestingly, among people with T2D and long‐term usage of DPP‐4is as a second‐line antidiabetic drug, a decreased fracture risk, including a decreased risk of upper arm fractures, was observed for a period of up to 5 years compared to those without DPP‐4i use.^(^ ^73^ ^)^

Comparison of various antidiabetic medications on fracture risk reveals divergent and intercontinental differences. A cohort study on T2D people observed no statistically significant increase in fracture risk with SGLT2i use compared with DPP‐4i use after 1 year.^(^ ^74^ ^)^ Additionally, another study found that treatment with metformin (hazard ratio [HR] of 0.88 [95% CI: 0.85 to 0.92]) and DPP‐4is (HR of 0.93 [95% CI: 0.88 to 0.98]) was associated with a reduced fracture risk, while insulin (HR of 1.26 [95% CI: 1.21 to 1.32]), thiazolidinediones (HR of 1.23 [95% CI: 1.18 to 1.29]), and meglitinides (HR of 1.12 [95% CI: 1.00 to 1.26]) were associated with an increased risk (*p* value <0.05).^(^ ^75^ ^)^ However, a Japanese study reported that both insulins, alpha‐glucosidase inhibitors, and DPP‐4i use were related to increased hip fracture risk compared to metformin. The risk of vertebral fractures was higher in people prescribed insulin, thiazolidine, and DPP‐4is compared with metformin.^(^ ^76^ ^)^ In contrast, another Japanese study reported a reduced fracture risk among those exposed to DPP‐4is.^(^ ^77^ ^)^ A Danish retrospective cohort study did not observe a significantly different risk of osteoporosis‐related fractures between people exposed to DPP‐4i,^(^ ^27^ ^)^ while a Taiwanese cohort study reported that the risk of osteoporosis was significantly lower among people exposed to DPP‐4is compared with those without DPP‐4i treatment (HR of 0.616 [95% CI: 0.358 to 0.961; *p* = 0.011]). Kaplan–Meier analysis showed that the preventive effect on osteoporosis was positively correlated with the cumulative dose of DPP‐4i (log‐rank, *p* = 0.039)^(^ ^78^ ^)^; although no fracture outcomes were reported, these findings may be relevant as the fracture risk seems to increase when T2D and osteoporosis coexist.^(^ ^79^ ^)^ A Korean study reported that initiating an SGLT2i was not linked with increased fracture risk compared to DPP‐4is.^(^ ^80^ ^)^ This was backed by another Korean study reporting no difference in fractures between DPP‐4i users and nonusers.^(^ ^81^ ^)^ Similarly, another study found that initiation of a SGLT2i versus a DPP‐4i was not associated with a higher risk of fractures regardless of estimated glomerular filtration rate (eGFR) among people with renal impairment.^(^ ^82^ ^)^ In agreement with the two aforementioned studies, an American study did not report differences in fracture risk between users of SGLT2is versus DPP‐4is.^(^ ^83^ ^)^ Lastly, a cohort study reported that the SGLT2i empagliflozin was associated with a similar risk of fractures compared to the use of DPP‐4is.^(^ ^84^ ^)^ No discrepancies in fracture risk were reported between users of DPP‐4is and SGLT2is in a large cohort study, and similar findings were reported by another study.^(^ ^85^, ^86^ ^)^

Lastly, it has been reported that significantly fewer falls and fractures occurred with linagliptin treatment compared to the sulphonylurea (SU) glimepiride.^(^ ^87^ ^)^ However, glimepiride may per se be associated with hypoglycemia and thus increase the risk of falling. In contrast to these findings, another study reported that DPP‐4is were not associated with an increased risk of fragility fractures compared with SU or insulin; however, they were associated with a lower risk versus thiazolidinediones.^(^ ^88^ ^)^

Overall, several meta‐analyses^(^ ^70^, ^71^ ^)^ and cohort studies^(^ ^27^, ^74^, ^75^, ^76^, ^77^, ^78^, ^81^, ^84^, ^85^ ^)^ demonstrated that DPP‐4is were either decreased or not associated with an increased risk of fractures.^(^ ^70^, ^71^ ^)^ Only one study reported an increase risk.^(^ ^76^ ^)^ Additionally, subgroup analyses showed that certain specific DPP‐4is, such as alogleptin^(^ ^72^ ^)^ and sitagliptin,^(^ ^70^, ^72^ ^)^ were associated with a reduced risk of fractures. Furthermore, intercontinental differences were observed in the association between antidiabetic medications and fracture risk. However, it is important to note that factors such as study design, population characteristics, and medication dosage may contribute to the variations in findings.

#### Bone turnover and bone structure

##### Human studies

This section includes three studies examining the effects of DPP‐4is on bone health in people with T2D.

*Effects of vildagliptin on bone markers*. A RCT investigated the effects of vildagliptin, a DPP‐4i, on bone markers.^(^ ^89^ ^)^ The study found that 1 year of vildagliptin exposure did not result in any significant changes in postprandial serum CTX concentrations (a marker of bone resorption) compared to pretreatment levels. Similarly, fasting serum ALP, calcium, and phosphate were also unaffected by 1 year of vildagliptin treatment. These results suggest that vildagliptin did not have a substantial impact on bone resorption markers or bone metabolism in the studied population.

*Association of plasma DPP activities with bone health*. A human study reported that elevated plasma DPP activities were associated with a higher proportion of osteoporosis/osteopenia in people with newly diagnosed T2D.^(^ ^90^ ^)^ While not directly linked to DPP‐4i use, this finding suggests a potential relationship between DPP activity and bone health in people with T2D. Elevated DPP activity may be an indicator of underlying mechanisms that could influence bone health, warranting further investigation.

Trabecular Bone Score (TBS) and BMD in DPP‐4i users was estimated by a retrospective study with T2D.^(^ ^91^ ^)^ They found that exposed to DPP‐4is had a higher TBS which is an index of bone microarchitecture. However, the BMD increased in both DPP‐4 exposed and unexposed groups, with no significant differences between the groups. The study suggests that DPP‐4i use might be associated with improved trabecular bone microarchitecture, but it did not show a significant difference in BMD between DPP‐4i users and non‐users.

Overall, the findings from these studies suggest that DPP‐4i use might have some effects on bone health in people with T2D, but the evidence is still limited and inconclusive.

##### Animal studies

This section includes several animal studies investigating the effects of various DPP‐4is on bone health in different diabetic animal models.

The effects of linagliptin on bone health were observed in diabetic mice. In a mouse study using a high‐fat diet to induce diabetes, impaired bone microarchitecture, reduced BMD, and altered bone turnover biomarkers were observed.^(^ ^92^ ^)^ Treatment with linagliptin alone and in combination with metformin significantly improved bone architecture, BMD, and bone turnover biomarkers. However, metformin alone did not show significant improvement in bone health. The results suggest that linagliptin, especially when used in combination with metformin, positively modulates bone health in diabetic mice.

The effects of vildagliptin, pioglitazone, and sitagliptin on bone health were studied in diabetic rats. A 5‐week study in male Zucker Diabetic Fatty (ZDF) rats used different treatment groups, including vildagliptin, pioglitazone, and their combination.^(^ ^92^ ^)^ The pioglitazone group showed decreased osteocalcin levels and increased TRACP 5b, indicating impaired bone metabolism. The results suggest that pioglitazone adversely affects bone health in diabetic rats.

A study using Wistar rats reported that sitagliptin‐treated diabetic animals had significantly lower serum levels of CTX‐I (a bone resorption marker) compared to untreated diabetic animals.^(^ ^93^ ^)^ μCT analysis showed that sitagliptin prevented cortical bone growth stagnation in diabetic rats, resulting in stronger femora during three‐point bending. However, another study in T1D rats reported that sitagliptin administration did not reverse the negative effects of T1D on bone indices, such as trabecular number and thickness.^(^ ^94^ ^)^ Additionally, the study on ZDF rats reported that vildagliptin treatment significantly increased BMD and trabecular bone volume. The combination therapy restored BMD, trabecular bone volume, and trabecular bone thickness, which were otherwise decreased by pioglitazone alone.^(^ ^95^ ^)^ Furthermore, the study using Wistar rats reported sitagliptin prevented cortical bone growth stagnation in diabetic rats assessed by micro–CT, resulting in stronger femora during three‐point bending.^(^ ^93^ ^)^

Lastly, the effects of DPP‐4i co‐administration with LPS in mice was studied and resulted in a lower osteoclast number and decreased bone resorption compared to LPS administration alone.^(^ ^96^ ^)^ This suggests that DPP‐4i may have a protective effect on bone resorption during inflammatory conditions.

Overall, animal studies provide valuable insights into the effects of various DPP‐4is on bone health in different diabetic animal models. Linagliptin,^(^ ^92^ ^)^ especially when used in combination with metformin, showed positive effects on bone health, improving bone architecture and turnover. Sitagliptin showed mixed results.^(^ ^92^, ^93^, ^94^ ^)^

#### Cell studies

A combined in vitro and in vivo rat study in diabetes induced by a high‐fat diet and streptozotocin reported that cell growth was disturbed while both the abnormal macrophage polarization and the endothelial impairment in diabetes were significantly alleviated by sitagliptin.^(^ ^97^ ^)^ Additionally, DM animals showed angiogenesis inhibition and poor bone formation on the bone–implant interface, which were significantly ameliorated by sitagliptin treatment.

To summarize, the consensus from most studies suggests that the use of DPP‐4is does not increase the risk of fractures. Some studies observed slight decreases in fracture risk with certain subtypes of DPP‐4is, although differences exist. However, it's important to consider that the choice of comparator drugs, the duration of follow‐up, and the specific indications for prescribing the drug being compared to the comparator might influence the findings.

## Discussion

### GLP‐1‐RAs

In general, the studies included in this review indicate that GLP‐1 RAs have a neutral to positive effect on bone health in people with and without T2D, whether for blood glucose regulation or weight loss therapy. Most of the presented studies describe a potential positive impact on bone health, as evidenced by various correlations observed in cell and animal studies. However, it should be noted that a definitive causal relationship demonstrating positive effects on bone health in humans has yet to be established.

**Fig. 2:**
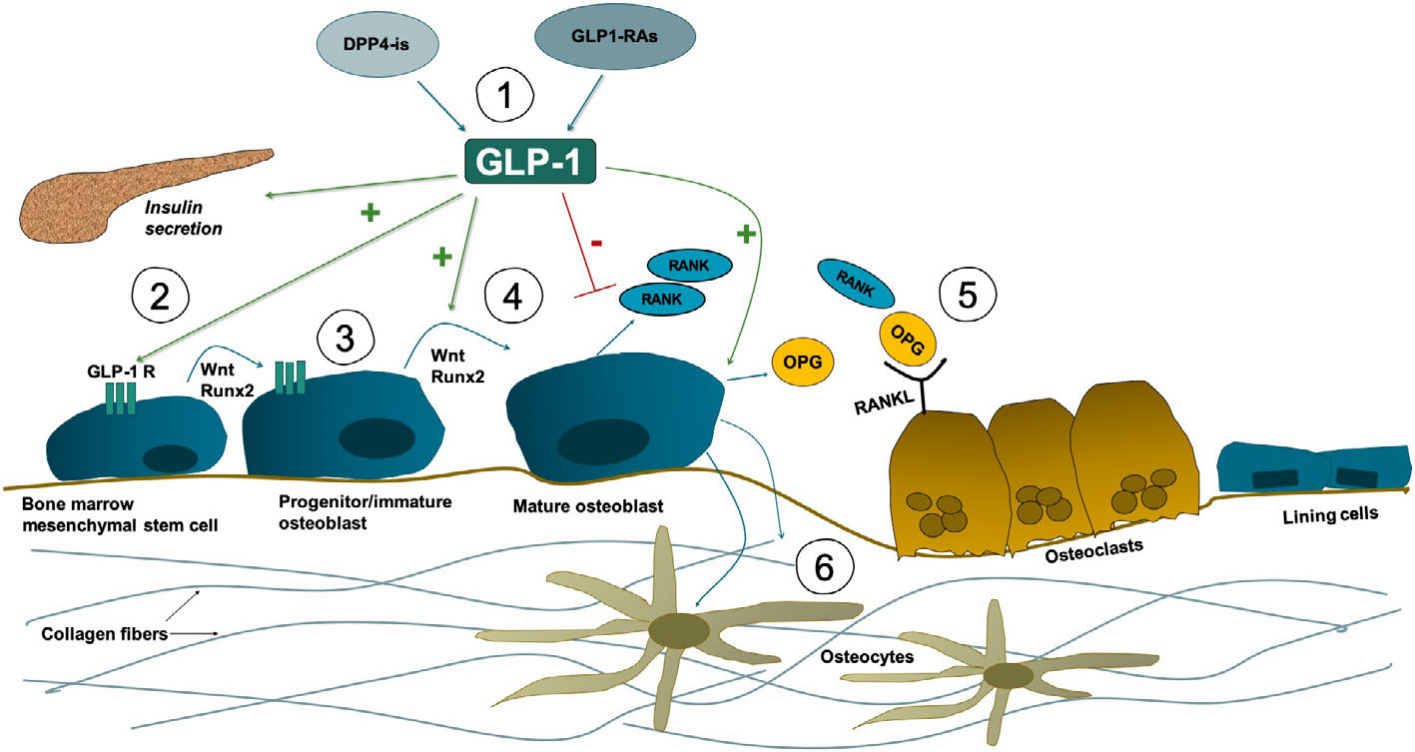
Potential mechanistic incretin‐mediated bone formation. (1) Dipeptidyl peptidase 4 inhibitors (DPP‐4i) and glucagon‐like peptide 1 receptor agonist (GLP‐1 RA) administration results in increased GLP‐1 activity. (2) GLP‐1 increases insulin secretion, resulting in anabolic effects on bone. (3) GLP‐1 binds to receptors on premature osteoblasts and stem cells inducing osteoblast proliferation. (4) This increases Runx2 gene expression (among others), resulting in activation of Wnt pathway and osteoprotegerin (OPG)/Receptor Activator of NF‐kB (RANK)/RANK Ligand (RANKL) ratio. (5) OPG acts as decoy receptor on RANKL, decreasing osteoclast activity and bone resorption. (6) In diabetes, GLP‐1 may have potential to restore imbalance in collagen crosslinks by changing phosphate/amide ratio.

GLP‐1‐RAs might prevent bone loss during weight loss assessed by evaluation of bone turnover markers, bone mass, and bone microstructure. The mechanism behind the positive effects of GLP‐1 on bone has been discussed widely in several systematic and narrative reviews. There have been suggestions of a direct effect on osteoclasts and osteoblasts through intracellular signaling pathways^(^ ^98^, ^99^ ^)^ or indirect effect on bone cells through thyroid c cells and calcitonin‐dependent inhibition of bone resorption.^(^ ^100^ ^)^ Moreover, a potential incretin‐mediated decrease of the chronic inflammation in T2D, which is believed to negatively modify bone tissue by nonenzymatic glycosylation, mineralization imbalance, and bone microdamage, has been suggested.^(^ ^99^ ^)^ See Figure 2 for an overview of the cellular mechanisms. However, human studies including T2D people are scarce, and this includes diverse endpoints, making it difficult to compare findings and draw reasonable conclusions. In T2D, BMD measurements do not sufficiently predict low bone quality. The clinical presentation of bone fragility is fractures, which are therefore, the most favorable outcome measure in people with T2D. No detrimental effects with GLP‐1 RAs were reported on the risk of fractures, although results were inconsistent, with heterogeneity between studies regarding people, study time, and comparison drugs. However, fracture assessment requires long‐term follow‐up, making it challenging to conduct intervention studies for assessment. Consequently, more sophisticated methods have emerged, i.e., HR‐pQCT and microindentation. Several studies reported a positive effect on trabecular bone microstructure but no effect on cortical bone. However, these studies were primarily on rodents without T2D. It has been suggested that people with T2D have a compromised cortical bone structure expressed as a higher cortical porosity, making these results even more challenging to interpret.^(^ ^101^, ^102^ ^)^ Human trials that include people exposed to GLP‐1 RAs have suggested that GLP‐1 may prevent an otherwise expected decrease in hip BMD during significant weight loss. However, only one of the included studies evaluated microarchitecture by HR‐pQCT, and no differences were observed. None of the identified human studies evaluated bone strength by microindentation. Although results were inconsistent, most animal studies suggested an improved or no effect on bone strength after GLP‐1 RA therapy. Collectively, human studies of bone health in response to GLP‐1 RA treatment are limited and trials including T2D people are warranted. As the validity of BMD and the cut‐off value for the osteoporosis diagnosis with a T‐score of −2,5 SD has been questioned in people with T2D, we propose that state‐of‐the‐art future research be conducted to focus on the assessment of bone indices using more advanced techniques, e.g., HR‐pQCT and microindentation.

However, in addition, studies that differentiate between different phenotypes of T2D with a focus on hard endpoints like fracture risk, fracture localization, and potential risk reduction during longer‐term follow‐up. Additionally, it is worth noting that not everyone may experience the beneficial effects of GLP‐RA therapy (approximately 10% to 15%), while others may experience significant and rapid weight loss and improved blood glucose control (in the case of T2D only), which raises questions about whether similar effects on bone health are maintained.^(^ ^103^ ^)^

Furthermore, most studies on GLP‐1 RAs and weight loss are conducted in conjunction with exercise and dietary advice. Exercise has a positive effect on weight maintenance by preserving muscle mass and overall benefits bone tissue.^(^ ^104^ ^)^ Therefore, robust evidence is needed regarding the impact on body composition. Moreover, the specific doses or dose–response effects of GLP‐1 RAs on bone health have not been thoroughly investigated, especially when considering the higher dosages used for weight loss compared to the treatment of T2D.

New therapies involving incretin hormones are emerging, such as combinations of GLP‐1‐RA and GIP or even with glucagon.^(^ ^105^, ^106^, ^107^ ^)^ Consequently, the modern‐day treatment approach utilizing incretin hormones for both weight loss and T2D is still in its early stages. Therefore, this topic is complex, and the current evidence supports a neutral or even a positive effect of incretin hormones on bone health. The crucial question that remains is whether GLP‐1 RAs represent a novel treatment for osteoporosis or merely a supplementary option for future strategies.

### DPP‐4is

DDP4is exhibit effects similar to those of GLP‐1 RA therapy but are considered less effective at regulating blood sugar levels and have fewer side effects due to their indirect mechanism of action. It is important to note that DPP‐4is are only prescribed to people withT2D and are not approved for weight loss. They are typically used in combination with other antidiabetic drugs and are rarely prescribed as monotherapy. Consequently, GLP‐1 RA drugs are generally regarded as superior to DPP‐4is.

Existing evidence indicates that DPP‐4is are neutral for bone health, as they do not increase the risk of fractures and may even have some positive effects on bone structure. However, further human studies are necessary to gain a clearer understanding of the impact of DPP‐4is on bone turnover, bone structure, and fracture risk. Future research should include longer‐term follow‐up and comparisons with other antidiabetic medications to better comprehend the role of DPP‐4is in promoting bone health.

Although DPP‐4is have their place in the treatment of T2D, their potential as a weight loss drug or their overall efficacy should be considered limited when compared to the numerous beneficial effects of GLP‐1 RAs and potential future combinations.

## Conclusion

Based on this systematic review, existing evidence is yet insufficient to support a positive or superior effect on bone health to reduce fracture risk in people with T2D. Collectively, incretin treatment is a potential therapeutic strategy for fracture prevention in people with T2D, but further evaluation is needed. Acknowledgement of T2D as an independent risk factor for osteoporosis‐related fractures, and the potential beneficial effects of incretins could encourage future T2D guidelines to include fracture risk assessment in the treatment algorithm for the choice of glucose‐lowering drug. However, further, larger, and more thorough clinical trials are necessary to investigate changes in bone indices and fracture risk after incretin treatment to confirm any potential advantageous effects on bone health in people with T2D.

## Perspectives

Ideally, large‐scale RCTs are needed, focusing on fractures as primary outcomes or using BMD as a proxy measure. Many existing RCTs did not prioritize fractures as the main outcome and had limitations in terms of duration, which may not capture changes in fracture risk adequately. Additionally, considering the diverse patterns of fracture risk in T2D and its different phenotypes, it becomes necessary to study subtypes of fractures rather than overall fracture risk. The interaction between T2D type and fracture risk also needs exploration, as factors such as body weight, insulin, insulin resistance, and metabolic control could potentially modify fracture risk. Moreover, reports on BMD from existing RCTs are scarce. However, considering that the excess fracture risk in T2D is limited and declining, conducting large‐scale RCTs with fractures as primary outcomes might not be feasible. Instead, studies should preferably focus on high‐risk groups, including people with prior fractures, low BMD, and severe insulin resistance, for example, as well as those with poor metabolic control.

Furthermore, there is a need for research investigating the impact of incretin‐mediated effects on bone health and loss in obese and overweight people. This area of study holds particular interest because bariatric surgery has been associated with an increased risk of fractures.^(^ ^108^, ^109^ ^)^

## Author Contributions

**Nicklas Højgaard‐hessellund Rasmussen:** Conceptualization; data curation; investigation; methodology; validation; visualization; writing – original draft; writing – review and editing. **Rikke Viggers:** Conceptualization; data curation; investigation; methodology; validation; writing – original draft; writing – review and editing. **Peter Vestergaard:** Conceptualization; investigation; methodology; project administration; supervision; writing – original draft; writing – review and editing.

## Funding

Novo Nordisk Foundation, Denmark (Grant NNF18OC0052064).

### Peer Review

The peer review history for this article is available at https://www.webofscience.com/api/gateway/wos/peer‐review/10.1002/jbm4.10817.

## Disclosures

Peter Vestergaard is head of research in the Steno Diabetes Center North Denmark sponsored by Novo Nordisk Foundation. Nicklas H. Rasmussen holds shares in Novo Nordisk and receives lecture fees from Borheringer Englheim and travel expenses from UCB. Rikke Viggers is funded by Novo Nordisk Foundation, Denmark (Grant NNF18OC0052064) and holds shares in Novo Nordisk.
